# Real-time assay for monitoring membrane association of lipid-binding domains

**DOI:** 10.1016/j.ab.2008.02.016

**Published:** 2008-06-01

**Authors:** Emma Connell, Phillip Scott, Bazbek Davletov

**Affiliations:** MRC Laboratory of Molecular Biology, Hills Road, Cambridge CB2 OQH, UK

**Keywords:** C2 domain, Turbidity, Lipid membrane, Synaptotagmin

## Abstract

The C2 domain is a common protein module which mediates calcium-dependent phospholipid binding. Several assays have previously been developed to measure membrane association. However, these assays either have technical drawbacks or are laborious to carry out. We now present a simple solution-based turbidity method for rapidly assaying membrane association of single lipid-binding domains in real time. We used the first C2 domain of synaptotagmin1 (C2A) as a model lipid-binding moiety. Our use of the common dimeric glutathione-*S*-transferase (GST) fusion tag allowed two C2A domains to be brought into close proximity. Consequently, calcium-triggered phospholipid binding by this artificially dimerized C2A resulted in liposomal aggregation, easily assayed by following absorbance of the solution at 350 nm. The assay is simple and sensitive and can be scaled up conveniently for use in a multiwell plate format, allowing high-throughput screening. In our screens, we identified nickel as a novel activator of synaptotagmin1 C2A domain membrane association. Finally, we show that the turbidity method can be applied to the study of other GST-tagged lipid-binding proteins such as epsin, protein kinase C-β, and synaptobrevin.

The C2 domain is a calcium-dependent phospholipid-binding module, first characterized in protein kinase C and subsequently identified in over 100 metazoan proteins [1–5]. It is particularly common in proteins involved in signal transduction or membrane trafficking. To study C2 domain membrane association several biochemical and biophysical methods have previously been developed using liposomes of defined composition. High-speed centrifugation to pellet the liposomes, with quantification of the amount of C2 protein associated with the pellet, is one such commonly used assay [6–8]. However, this has the caveat that any protein aggregates formed in the presence of calcium may also pellet, giving anomalously high levels of apparent protein/lipid association [9].

Another method involves use of glutathione-*S*-transferase (GST)^1^-tagged proteins immobilized on glutathione beads, with the amount of radioactive or fluorescent liposomes bound to the bead pellet being quantified [6,8,10–12]. Although these assays have the advantage of avoiding high centrifugation speeds, they are laborious and time consuming, requiring attachment of protein to beads, aliquoting of these beads (which is intrinsically imprecise), incubation with liposomes, and subsequent bead washing with low-speed centrifugations. Another clear disadvantage of both of the above methods is that it is not possible to follow membrane association in real time. A further technique involves labeling the C2 domains themselves, for example fluorescently or with a spin label for electron paramagnetic resonance studies. This labeling often requires mutagenesis of the protein to introduce a functional group such as SH sulfhydryl (present in cysteine) for tag attachment [13–16]. This may also introduce problems such as inactivation of the protein or artificial enhancement of lipid binding due to the introduction of hydrophobic tags. A major disadvantage of all of the above methods is the requirement for high-cost equipment such as ultracentrifuges, fluorimeters, scintillation counters, or electromagnets capable of generating strong magnetic fields.

We investigated the possibility of detecting C2 domain membrane interaction in solution, without protein modification, by employing conventional spectrophotometry. An increase in particle size, which occurs upon liposome cross-linking, can be detected by following solution turbidity. We reasoned that, by expressing a C2 domain attached to a dimeric fusion tag, we could artificially bring two C2 domains into close proximity. Because each individual C2 domain binds one liposomal membrane the dimeric nature of the tag could result in the adjoined C2 domains cross-linking liposomes, thus producing an increase in turbidity. We considered several common fusion tags used for recombinant protein expression, such as GST, maltose-binding protein, and His tag, and decided upon GST because this is known to be dimeric [17,18].

Here, we present a turbidometric assay which avoids both centrifugation and protein modifications. Using the GST tag it is possible to determine the degree of liposome binding of a single lipid-binding domain by monitoring solution turbidity. We used the first GST-tagged C2 domain of synaptotagmin1, C2A, to validate the assay [10,19,20]. A rapid increase in solution absorbance at 350 nm was observed upon mixing of GST-C2A with liposomes in the presence of calcium, indicative of an overall increase in particle size (liposome aggregation). This solution-based method is quick and simple, requiring only a basic laboratory spectrophotometer. It is also possible to make kinetic measurements of membrane/protein association in real time. Using this assay, we show that the C2A domain of synaptotagmin1 binds membranes in a cooperative and electrostatic manner and that it is sensitive to the presence of several divalent cations, including nickel. We also demonstrate that this assay can be used for other lipid-binding proteins and can be scaled up for large-scale, high throughput screening.

## Materials and methods

### Recombinant protein production

GST fusion constructs of synaptotagmin1 C2A wild-type and mutants, protein kinase C-β C2 domain, ENTH domain of epsin, and the cytoplasmic domain of synaptobrevin (residues 1–96) were described previously [10,21–23]. Expression of recombinant protein fragments was induced by addition of 0.1 mM isopropyl β-D-thiogalactopyranoside to 1 liter cultures of BL21 *Escherichia coli* cells before incubation overnight at 19 °C. After pelleting, the bacteria were resuspended in 12 ml 0.5 M NaCl, 20 mM Hepes, pH 7.3, 1 mM dithiothreitol, 1 mM ethylenediaminetetraacetic acid (EDTA) before lysis with an Emulsiflex homogenizer (Avestin, Canada) and further solubilization by addition of 2% Triton X-100 detergent. The soluble portion of the lysate (containing approximately 30 mg of the protein of interest) was added to glutathione Sepharose beads (2 ml bead volume; GE Healthcare, UK) and incubated (1.5 h, 4 °C) to capture GST-tagged protein. The beads were then thoroughly washed once in high-NaCl (1 M) and three times in low-NaCl (0.1 M) buffers containing 20 mM Hepes, pH 7.3, 1 mM EDTA, and 0.1% Triton X-100.

GST-tagged protein was eluted from the beads with two successive rounds of incubation (30 min, 4 °C), each with 6 ml of glutathione elution buffer comprising 15 mM glutathione, 0.25 M NaCl, 0.1% Triton X-100, and 20 mM Hepes, pH 8.5. Eluted proteins were concentrated using Vivaspin 10,000 molecular weight cut-off protein concentrators (Sartorius Group, UK) and further purified by gel filtration on a Superdex 200 column (GE Healthcare, UK), which was equilibrated in 0.1 M NaCl and 20 mM Hepes, pH 7.3 (buffer A). Gel filtration was carried out at a flow rate of 0.5 ml/min and the fractions containing protein (detected by their absorbance at 280 nm) were collected and analyzed by SDS–PAGE and Coomassie staining.

For purification of untagged C2A and synaptobrevin, the GST-tagged proteins were isolated from bacterial extract by binding to glutathione beads as above, with identical bead washing. After a further three washes with buffer A they were incubated (30 min, 37 °C) in buffer A with 100 units of thrombin to remove the GST tag. Cleavage was checked using SDS–PAGE and Coomassie staining. Protein concentration and gel filtration were then carried out as above.

### Liposome preparation

Chloroform solutions of brain phosphatidylcholine, phosphatidylserine, phosphatidylethanolamine, phosphatidylinositol-4,5-bisphosphate, and cholesterol (Avanti Polar Lipids, US) were mixed in the stated ratios and dried under nitrogen for 30 min. Dried lipids were resuspended in 0.1 M NaCl, 20 mM Hepes, pH 7.3, and 2 mM EDTA (buffer B) to form large multilamellar vesicles at 2.5 mg/ml. These were extruded with 20 passes through a polycarbonate Nucleopore Track-Etch membrane (Whatman, UK), producing unilamellar vesicles of the required size. Lipid recovery after extrusion was approximately 90% and the size of the liposomes was relatively homogeneous (confirmed using dynamic light scattering).

### Turbidity assays

Absorbance measurements were performed at 350 nm; this wavelength was chosen because it gave the best signal to noise ratio in the 350- to 500-nm range (data not shown; lower wavelengths were not investigated because protein itself absorbs at 280 nm). The change in absorbance indicates a change in turbidity of the liposomal solution, corresponding to increased particle size (aggregation). Experiments were carried out either in disposable UV-compatible plastic cuvettes (Eppendorf, UK) in 100 μl final volume for kinetic measurements using a Genesys 6 spectrophotometer (Thermo Electron Corp., UK) or in a 96-well transparent plate format (Corning, US) in a 60-μl final volume for screening using a plate reader (Tecan, Switzerland). The absorbance values obtained with the plate reader were typically 2.5-fold lower than measurements using the Genesys spectrophotometer, due to a difference in equipment sensitivity. Despite this lower sensitivity, the obvious advantage of the plate reader is that it allows rapid, nearly simultaneous acquisition of multiple absorbance values. For kinetic measurements, we typically used the more sensitive, single-cuvette spectrophotometer. All reactions were carried out at 22 °C in buffer B. Calcium was added at 3 mM to give a final free calcium concentration of 1 mM. For the calcium titration experiments, added calcium and EDTA concentrations were determined as in [10].

In the case of the single cuvette measurements, 20 μl liposomes (2.5 mg/ml) were premixed with 40 μl buffer B and absorbance was then read every second. After 30 s, calcium solution (20 μl) was added and, following a further 30 s, 20 μl of protein was also added. For the multiwell 60-μl reactions, volumes were scaled down proportionally. Small drops in turbidity immediately upon addition of calcium or protein are consistent with dilution of the reaction mix by 20–25%, and the reactions were mixed at the point of each addition by brief pipetting. Prism4 (GraphPad, USA) was used for statistical analysis. Error bars represent the standard deviation of triplicate readings.

### Dynamic light scattering

Dynamic light scattering measurements were performed using a particle size analyzer (Zetasizer NanoS, Malvern Instruments, UK) with a 4-mW He-Ne laser at a wavelength of 633 nm. Particle sizes were obtained at a back-scattering angle of 173°, to reduce anomalous multiple scattering and dust particle scattering contributions. The total reaction volume was 100 μl (mixed as above) and the measurements were started after 2-min incubation at 22 °C. The run time was typically 3 min.

### GST-C2A bead binding assay

Liposomes were prepared as above but with addition of the hydrophobic dye DiO (Invitrogen, UK; 10 μg dye for 1 mg lipid) before drying the lipids under nitrogen. GST-C2A attached to glutathione beads (15 μl bead volume with 10 μg bound GST-C2A per reaction) was incubated with these fluorescent liposomes (0.5 mg/ml) in the presence of various concentrations of free calcium [10]. Incubations were carried out in 100-μl total volumes with constant shaking (130 rpm, 30 min, 22 °C) to keep the beads suspended in solution. The beads were then gently pelleted (2 min, 400 g) and washed three times with 1 ml of the relevant calcium incubation buffer (containing the same concentration of free calcium as in the reaction). The bead pellet was resuspended in 300 μl buffer A containing 0.1% Triton X-100 to disrupt liposomes and the released fluorescence was quantified using a plate reader (excitation at 484 nm, emission monitored at 501 nm; Tecan, Switzerland).

## Results

### Turbidity measurements of GST-C2A membrane binding in presence of calcium

**Fig. 1:**
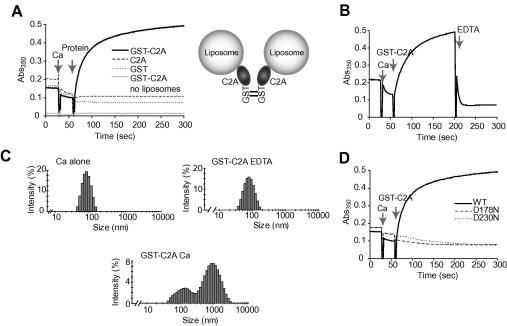
Aggregation of liposomes by GST-tagged synaptotagmin1 C2A. (A) Left: Graph showing absorbance changes of liposomal solution at 350 nm upon addition of 1 mM free calcium (Ca) and GST-C2A, GST, or C2A with time. Also shown is absorbance change of liposome-free solution with GST-C2A and calcium. Right: Schematic illustrating dimeric nature of GST tag bringing two C2A domains and thus two liposomes into close proximity. (B) Increase in absorbance driven by GST-C2A and calcium is reversible upon addition of a calcium chelator, EDTA (5 mM). (C) Dynamic light scattering confirms increase in particle size in the presence of GST-C2A and calcium, compared with calcium alone or GST-C2A with EDTA. (D) Mutations D178N or D230N disrupt the calcium-binding ability of the synaptotagmin1 C2A domain. GST-C2A carrying either of these mutations is unable to promote calcium-dependent liposome aggregation. Reactions in (A)–(D) contained phosphatidylcholine:phosphatidylserine liposomes, PC:PS (3:1) at 0.5 mg/ml and protein at 2.5 μM.

Initially, we measured the association of the synaptotagmin1 C2A domain with 80 nm liposomes over time, in the presence of calcium. The turbidity of liposome solutions was monitored before and after addition of calcium and either GST, C2A, or GST-C2A. Upon addition of calcium followed by GST-C2A, an immediate and robust increase in absorbance was observed (Fig. 1A, left). After 10–15 s half-maximal increase was attained. Neither untagged C2A nor GST in the presence of calcium was able to promote this increase in turbidity. In addition, GST-C2A and calcium in the absence of liposomes gave no turbidity increase. Thus, when brought into close proximity by the presence of the dimeric GST tag, two C2A domains can efficiently cross-link liposomal membranes in the presence of calcium (Fig. 1A, right). Fig. 1B shows that the increase in turbidity promoted by GST-C2A with calcium is reversible upon addition of the divalent metal ion chelator EDTA. This shows that the observed rise in absorbance is not due to liposome fusion but is instead attributable to reversible liposome aggregation. An independent technique, dynamic light scattering, confirmed that the increase in turbidity in the presence of GST-C2A and calcium indicates a genuine increase in particle size (Fig. 1C). Again, GST-C2A-mediated liposomal aggregation was calcium dependent, because it did not take place in the presence of EDTA.

**Supplementary Figure 1:**
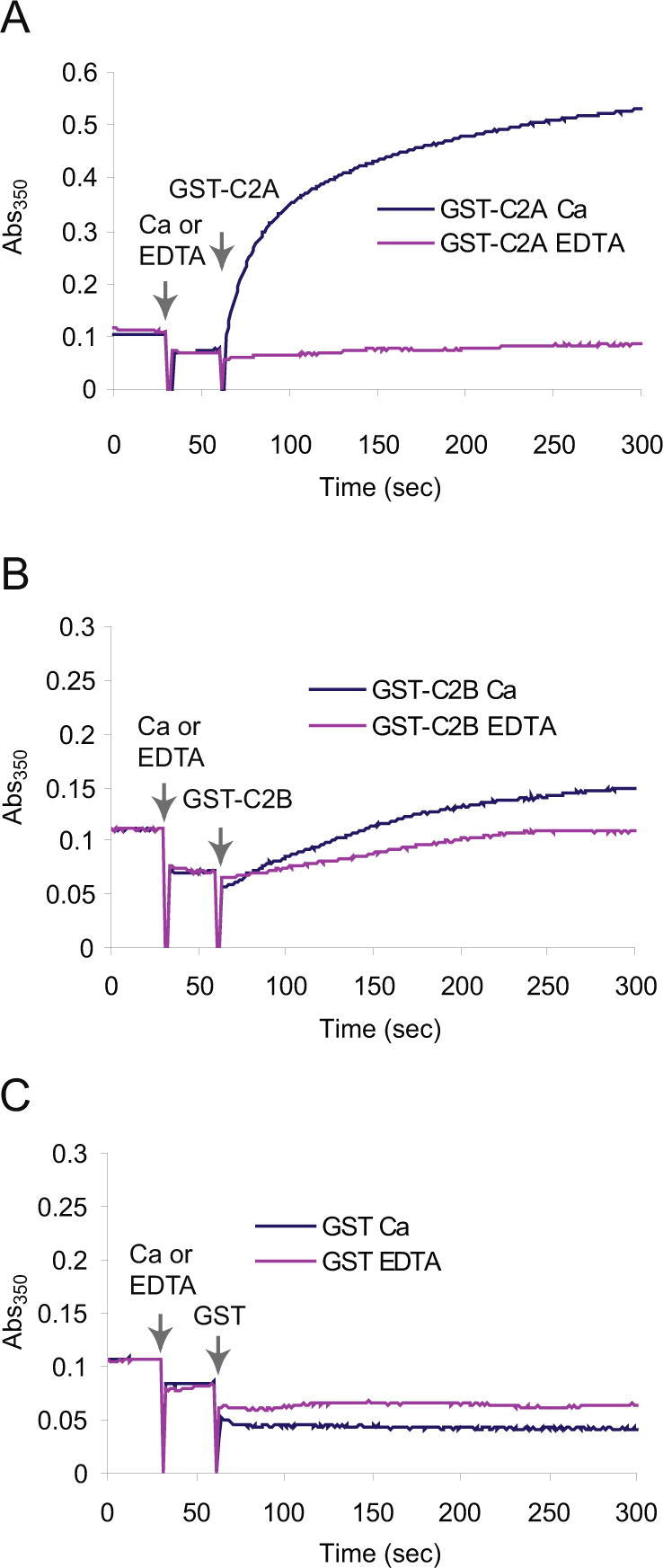
Direct comparison of phospholipid binding ability of the two individual C2 domains of synaptotagmin 1 by turbidity. Shown are the real-time absorbance traces of liposomal solutions upon addition of either (A) GST-C2A (B) GST-C2B or (C) GST alone in the presence or absence of 100 μM free calcium. Although GST-C2A binds membranes more efficiently than GST-C2B, turbidity assays can detect even this weaker C2B-liposome association. Liposomes are 0.5 mg/ml phosphatidylcholine:phosphatidylserine, PC:PS (3:1), protein is 2.5 μM, GST-C2B was purified as in Rickman *et al.* (2004) J Biol Chem **279**, 12574-12579.

To demonstrate that the turbidity increase is an intrinsic property of calcium-sensitive C2A, we tested GST-C2A proteins where the calcium-binding regions of the C2A domain were disrupted by mutation (D178N or D230N). These mutations abolish the calcium-dependent membrane aggregation ability of GST-C2A (Fig. 1D). We conclude that wild-type C2A, when artificially dimerized by GST, can specifically and rapidly aggregate liposomes in a calcium-dependent manner, as assessed by turbidity measurements in real time. Of note, our turbidity assay is also capable of monitoring the weaker binding of the second C2 domain of synaptotagmin1, C2B, to membranes (Supplementary Fig. 1) [24,25]. This has been difficult to detect using alternative assays [26,27].

### Influence of liposomal size on GST-C2A calcium-dependent turbidity increase

**Fig. 2:**
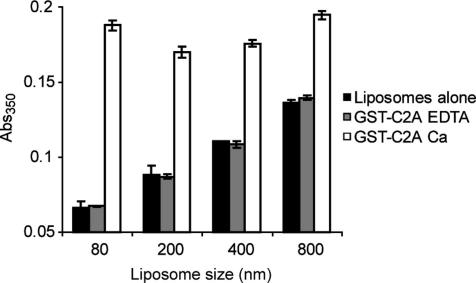
Dependence of the GST-C2A calcium-dependent turbidity signal on liposomal size. Reactions containing liposomes of increasing size, either alone or with GST-C2A ± calcium, were incubated for 15 min in triplicate in a multi well plate before measuring turbidity. Liposomes are 0.5 mg/ml PC:PS (3:1); free calcium is 1 mM; protein is 2.5 μM.

Since the above experiments were all carried out with 80-nm unilamellar liposomes it was interesting to determine whether protein-mediated liposomal aggregation could also be observed by turbidity with larger liposomes. Reactions were set up in triplicate in a 96-well plate format and incubated for 15 min to allow maximal liposomal aggregation to occur before reading absorbance using a plate-reader spectrophotometer. The multi well plate format allowed rapid end-point acquisition of multiple measurements at each liposome size. Fig. 2 shows that GST-C2A/calcium promotes an increase in turbidity for liposomes of various sizes. However, larger liposomes exhibit higher initial turbidity values and correspondingly lower increases with GST-C2A/calcium, resulting in suboptimal calcium-dependent turbidity signals. We conclude that liposomes with an extruded size of 200 nm or less can be effectively used in this assay.

### Characterization of C2A–membrane interaction

**Fig. 3:**
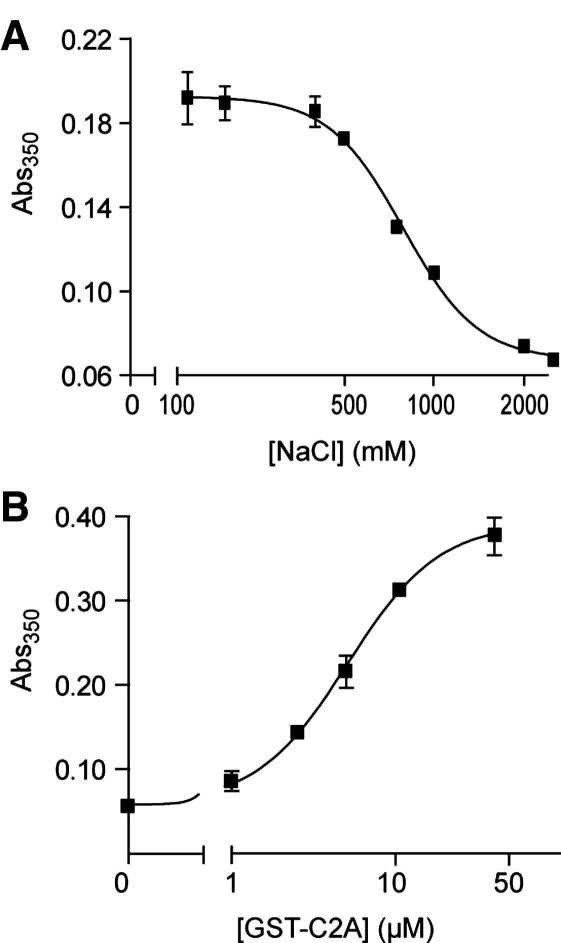
GST-C2A interaction with membranes is electrostatic and cooperative, as determined by the turbidity assay. (A) Turbidity measurements showing absorbance of liposomal solutions after 15-min incubation with GST-C2A (2.5 μM) and calcium, with various salt concentrations. There is an exponential decrease in liposome aggregation with increasing concentrations of NaCl. (B) Increasing turbidity of liposomal solutions after 15-min incubation with calcium and the indicated concentrations of GST-C2A. The best curve-fitting gave an EC_50_ value of 5.1 μM, and a Hill coefficient of 1.6. Liposomes are 0.5 mg/ml PC:PS; free calcium is 1 mM.

It is known that C2A binds membranes in an electrostatic manner [28]; therefore to further validate our assay we decided to carry out turbidity measurements in multiwell plate format with GST-C2A/calcium and liposomes in the presence of increasing concentrations of NaCl. Fig. 3A shows that, as the amount of salt increases, the ability of the C2A domain to bind membranes decreases exponentially, confirming that the C2A–phospholipid association is electrostatic in nature. We also tested liposomal aggregation with increasing concentrations of GST-C2A. A sigmoidal dose–response curve was obtained, with an EC_50_ of 5.1 μM and a Hill coefficient of 1.6, illustrating the cooperative nature of GST-C2A membrane association (Fig. 3B).

**Fig. 4:**
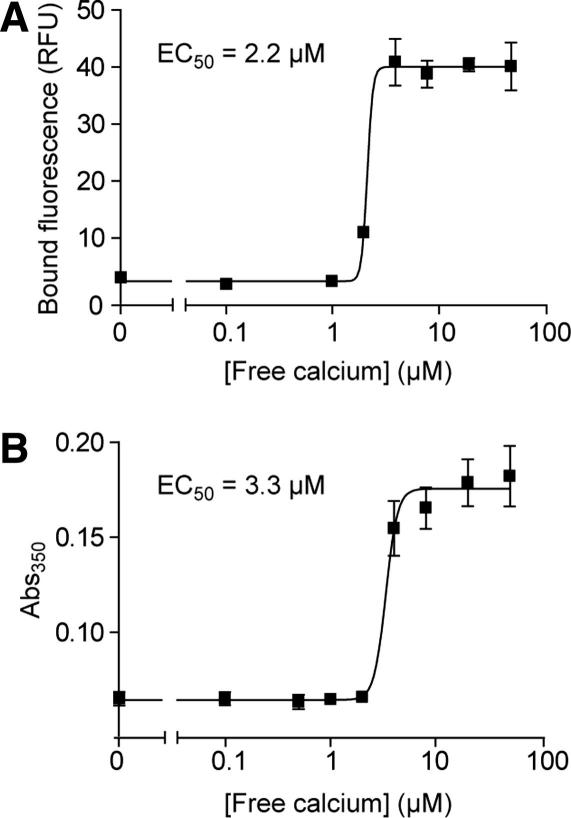
Comparison of apparent calcium sensitivity of C2A assessed by bead-binding and turbidity assays. (A) GST-C2A bound to glutathione beads was incubated with fluorescent liposomes and increasing concentrations of free calcium (30 min, 22 °C, with shaking). The bead pellet was then washed three times, and the amount of pellet-associated liposome fluorescence was quantified. RFU, relative fluorescence units. (B) Liposomes were incubated with GST-C2A and increasing concentrations of free calcium for 15 min at 22 °C, before measuring the absorbance of the solution at 350 nm using a plate-reader spectrophotometer. Liposomes are 0.5 mg/ml PC:PS; GST-C2A is 2.5 μM.

Next, we sought to directly compare the sensitivity of our absorbance-based assay with a standard bead-binding technique for measuring membrane association of a protein. We first carried out the bead-binding assays, incubating fluorescently tagged liposomes with GST-C2A attached to glutathione beads, in the presence of various concentrations of free calcium. Fig. 4A shows that upon raising the calcium concentration the amount of liposomes associated with the protein on the beads sharply increased exponentially, with an EC_50_ of 2.2 μM calcium, close to the previously published value [10]. However, as mentioned in the introduction, this assay is time consuming, taking on average around 5 h.

We then carried out turbidity assays in plate-reader format with the same batch of GST-C2A, liposomes, and calcium buffers for comparison. It took only 20 min to obtain the data and the EC_50_ calculated was almost identical to that obtained from the bead-binding assay (3.3 μM; Fig. 4B). Therefore, using a rapid solution-based assay, the nature of protein–membrane interactions can be easily and accurately detected.

### Rapid screening of C2A–membrane binding in the presence of various cations

**Fig. 5:**
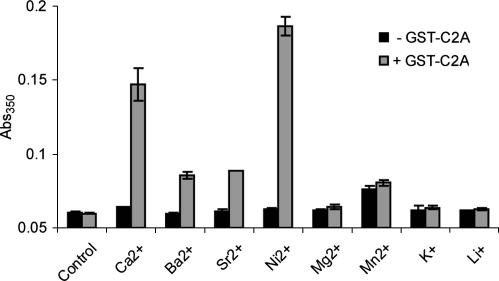
Cation screening of GST-C2A-mediated membrane aggregation. Liposomes (0.5 mg/ml PC:PS) were incubated (15 min) with various divalent and monovalent cations (1 mM, all chloride salts) with or without 2.5 μM GST-C2A. The plate-reader spectrophotometer was then used to measure absorbance.

We employed our multiwell turbidity assay to screen GST-C2A membrane association in the presence of various divalent and monovalent metal ions. Fig. 5 shows that, of the cations tested, calcium and nickel were the most effective at promoting GST-C2A membrane association. The result with nickel was unexpected and should be noted by those purifying His-tagged synaptotagmins using nickel-affinity chromatography. Barium and strontium were also capable of promoting vesicle aggregation with GST-C2A but to a lesser extent. The other divalent and monovalent cations were ineffective.

### Membrane-binding ability of other GST-tagged lipid-binding modules assessed by turbidity

**Fig. 6:**
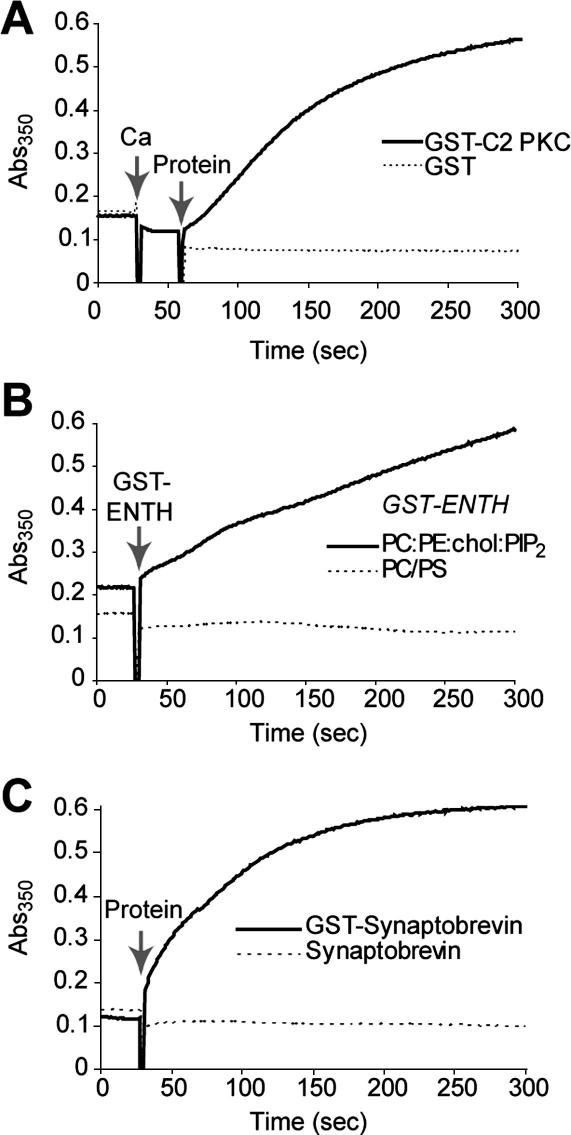
Turbidity measurements of liposome aggregation by diverse lipid-binding proteins. (A) Aggregation of PC:PS (3:1) liposomes by 2.5 μM GST-C2 domain of protein kinase C-β (C2 PKC) in the presence of calcium. GST alone does not cause aggregation. (B) Aggregation of PC:PE:Chol:PIP_2_ (4:4:1:1) liposomes with 10 μM GST-ENTH domain of epsin. GST-ENTH does not aggregate liposomes lacking PIP_2_. (C) Aggregation of PC:PS (3:1) liposomes by 10 μM GST-synaptobrevin (residues 1–96). Untagged synaptobrevin does not cause aggregation. PIP_2_, phosphatidylinositol-4,5-bisphosphate; Chol, cholesterol. Liposomes were 0.5 mg/ml; free calcium was 1 mM.

It was important to confirm that our assay could be applied to other lipid-binding proteins. We carried out turbidity assays with the calcium-sensitive C2 domain of protein kinase C-β (PKC) [29] and used two proteins which bind lipids in a different manner, the epsin N-terminal homology (ENTH) domain of epsin and the cytoplasmic domain of synaptobrevin [30]. In each case the GST tag was used to link two lipid-binding monomers. Fig. 6A shows that GST-PKC C2 domain promoted efficient cross-linking of PC:PS liposomes in the presence of calcium. GST alone was ineffective. Fig. 6B shows that GST-tagged ENTH domain was able to cross-link liposomes containing 10% phosphatidylinositol-4,5-bisphosphate (PIP_2_), an important cofactor for lipid binding of this domain [22]. Interestingly, the kinetics of this constitutive association is slower than that with calcium-sensitive C2 domains. As expected, GST-ENTH was unable to aggregate membranes which did not contain PIP_2_, showing that our assay can be used to decipher the lipid-binding preferences of a protein. Finally, Fig. 6C shows rapid GST-synaptobrevin aggregation of PC:PS membranes, consistent with earlier studies employing, for example, surface plasmon resonance [31–34]. Untagged synaptobrevin was not able to aggregate liposomes. We conclude that the turbidity-based kinetic assay is a useful and simple method for characterization of membrane-binding activity of a diverse range of lipid-binding domains.

## Discussion

We have presented a rapid and sensitive turbidometric assay for the study of GST-tagged lipid-binding proteins. Protein–membrane association can also be followed in real time. The calcium-dependent phospholipid-binding module GST-C2A from synaptotagmin1 was used as a model protein to illustrate the effectiveness and reproducibility of the method. GST-C2A aggregated unilamellar liposomes rapidly and reversibly in an electrostatic and cooperative manner and with an EC_50_ of 3.3 μM calcium. Our turbidity data are in good agreement with the previously published characteristics of C2A [10,28], thus validating the assay for future studies of protein–membrane association. Our assay was also sensitive enough to detect the weaker membrane binding of the synaptotagmin1 C2B domain (Supplementary Fig. 1). A similar turbidity method has previously been used in the study of calcium-dependent aggregation of membranes by oligomeric annexins [35]; however, as we have demonstrated here, it can clearly be applied to the study of other single lipid-binding modules if they are brought into close proximity by the presence of the protein purification tag, GST.

We have demonstrated that the synaptotagmin1 C2A domain is sensitive to nickel, barium, and strontium, in addition to calcium. The nickel-dependent strongly stimulating effect was unexpected and is important to note because His-tag protein purification protocols require immobilization on a nickel-affinity chromatography column. Any free nickel may promote the association of synaptotagmins purified in this manner with lipids.

In summary, our solution-based turbidometric approach to the study of membrane-binding proteins allows the activity of a single lipid-binding domain to be assayed by conveniently exploiting the dimeric nature of GST, a purification tag which is commonly used in many laboratories. The assay is rapid, reproducible, and highly sensitive and does not require expensive reagents or equipment. It can also be used to measure the kinetics of membrane association of a wide range of lipid-binding proteins.

## Footnotes

1 *Abbreviations used:* GST, glutathione-*S*-transferase; PKC, protein kinase C-β; ENTH, epsin N-terminal homology; PC, phosphatidylcholine; PS, phosphatidylserine; EDTA, ethylenediaminetetraacetic acid.
